# The improved business valuation model for RFID company based on the community mining method

**DOI:** 10.1371/journal.pone.0175872

**Published:** 2017-05-01

**Authors:** Shugang Li, Zhaoxu Yu

**Affiliations:** 1 School of Management, Shanghai University, Shanghai, PR China; 2 Department of Automation, East China University of Science and Technology, Shanghai, PR China; West Virginia University, UNITED STATES

## Abstract

Nowadays, the appetite for the investment and mergers and acquisitions (M&A) activity in RFID companies is growing rapidly. Although the huge number of papers have addressed the topic of business valuation models based on statistical methods or neural network methods, only a few are dedicated to constructing a general framework for business valuation that improves the performance with network graph (NG) and the corresponding community mining (CM) method. In this study, an NG based business valuation model is proposed, where real options approach (ROA) integrating CM method is designed to predict the company’s net profit as well as estimate the company value. Three improvements are made in the proposed valuation model: Firstly, our model figures out the credibility of the node belonging to each community and clusters the network according to the evolutionary Bayesian method. Secondly, the improved bacterial foraging optimization algorithm (IBFOA) is adopted to calculate the optimized Bayesian posterior probability function. Finally, in IBFOA, bi-objective method is used to assess the accuracy of prediction, and these two objectives are combined into one objective function using a new Pareto boundary method. The proposed method returns lower forecasting error than 10 well-known forecasting models on 3 different time interval valuing tasks for the real-life simulation of RFID companies.

## 1. Introduction

In recent years, driven by the rise of wireless sensor network[1][2], Internet of things[3], Radio Vehicular Networks [4], Cloud Systems[5] and other Cyber-Physical Systems[6], the development of radio frequency identification (RFID) industries has reached a new milestone. It makes the breakthrough progress in all areas of production and life, especially in the financial payment, retail supply chain, clothing supply chain, logistics, and product traceability. Those followed by the gradually expanding of RFID market. IDTechEx found that in 2015, the total RFID market was worth $10.1 billion, up from $9.5 billion in 2014 and $8.8 billion in 2013, and it will rise to $13.2 billion in 2020[7]. As a result, the appetite for funding for RFID technology is growing rapidly. According to PrivCo statistics, there were 23 well-known RFID-related companies’ mergers and acquisitions (M&A) in the three years from 2013 to 2015, and in 2014 alone, 10 cases of M&A deals in related industries occurred [8].

As business valuation tasks appear throughout time in M&A operation, they are often solved with dozens of different models, for instance payback period method, non-discounted approach, discount algorithm, net present value method, present value index method, real options approach (ROA)[9][10],etc. In business valuation system, profit forecasting is an important input for calculating value of the acquired company's worth. In M&A operation, buyers use profit forecasting for managing business evaluation and pricing strategies. Keeping a high profit forecasting accuracy is important due to low profit margins in the industry. It would be beneficial for all but especially for the buyers when they could have a solution that can play a role of expert for them and give them lowest profit forecasting error for all of business valuation tasks.

In general, profit forecasting is a difficult task as it is affected by a group of indicators which descript the conditions of the external and internal environment of the company. Holistic overview of the current literature indicates that profit forecasting methods are mainly divided into two categories. One is statistical methods, such as regression model (RM), time series analysis, and gray system theory, another is neural network (NN) method[11][12]. However, little attention has been directed to network graph (NG) method. To address these problems, we define NG to describe the non-linear relationship between the company's net profitability and its external and internal environment factors[13], and develop the enhanced community mining method (ECMM) based on Bayesian method (BM) for company profit predicting. ECMM successfully models the complex nonlinearity and gives better results than the traditional RMs and NN methods because ECMM can set flexible coefficients for different values of the independent variable and adopt complex nonlinear function to map the relationship between the independent variables and dependent variable. In ECMM, BM is adopted to express the clustering rules of NG’s nodes. Furthermore, the improved bacterial foraging optimization algorithm (IBFOA) is developed to calculate Bayesian probability function (BPF) as well as to optimize their parameters. IBFOA is distinctive because it generates new solution according to the statistical analysis of the existing solutions and adaptively adjusts its parameters, those can increase the diversity of the population and avoid the prematurity. Last but not least, we use bi-objective method to assess the accuracy of prediction and Pareto boundary method (PBM)[14][15] is created to integrate the two indicators into one objective function, as a result the reliability of the valuation model is improved.

Based on the predict result of the company’s future net profit by ECMM, the company value is assessed using ROA since it is preferred in valuing projects where uncertainty is high [16]. Simulation results of RFID companies in China show that the proposed method can significantly improve the accuracy and reliability of ROA.

The rest of paper is organized as follows: Section 2 presents the company valuation system. Section 3 provides ECMM in NG. In section 4, IBFOA is proposed. In section 5, experiments with real word data are used to demonstrate the workability of our proposed method. Section 6 offers the conclusions.

## 2. The business valuation system

**Fig 1:**
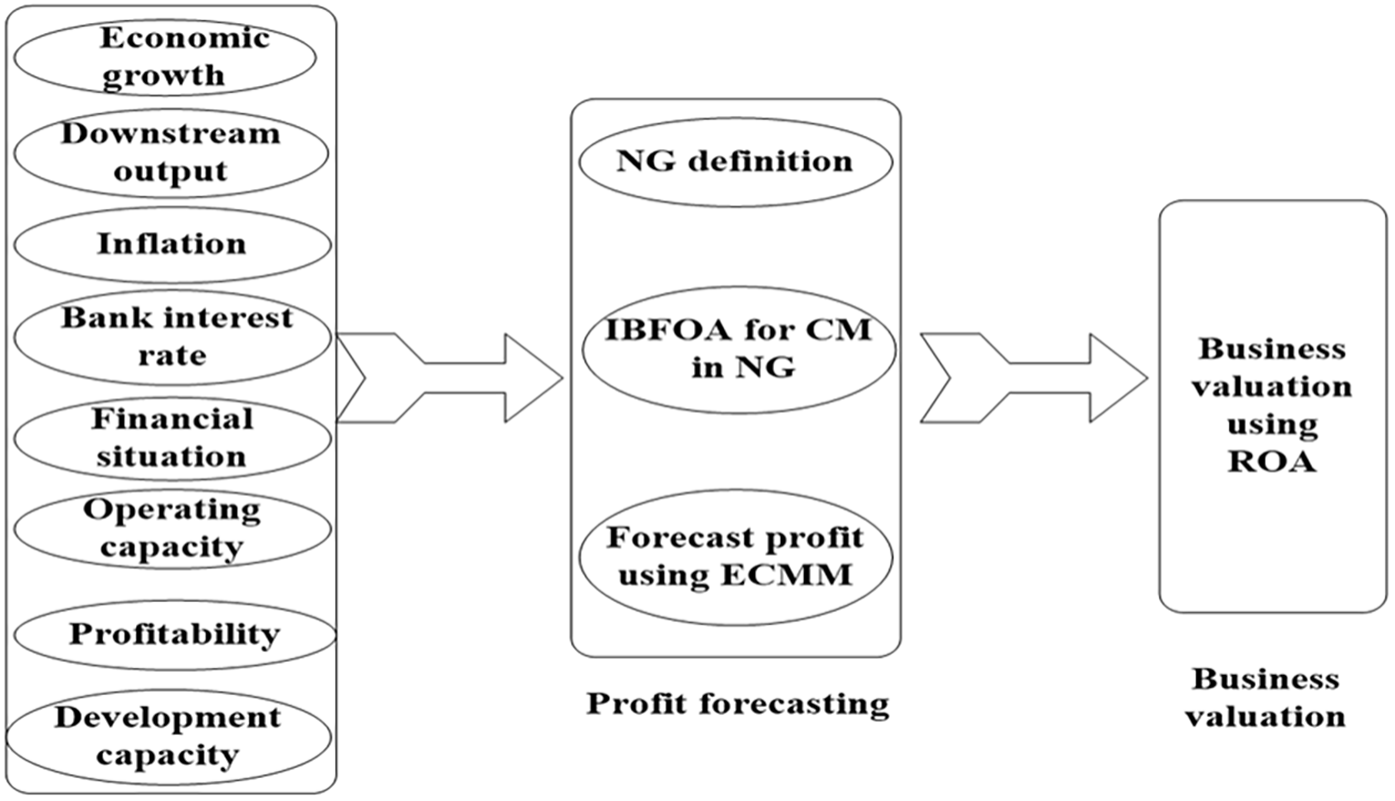
The business valuation system.

The proposed business valuation system of RFID companies is depicted in Fig 1. It consists of the following modules: key factors, profit forecasting, and business valuation. In the first module the key external and internal factors affecting the company profitability are identified. In the second module, IBFOA is designed to find communities in NG and ECMM is developed to forecast the company profit. Business valuation module assesses the value of RFID companies according to the forecast results of ECMM, which gives a full consideration on the investment opportunity value of the RFID companies.

### 2.1. Evaluation factors

**Fig 2:**
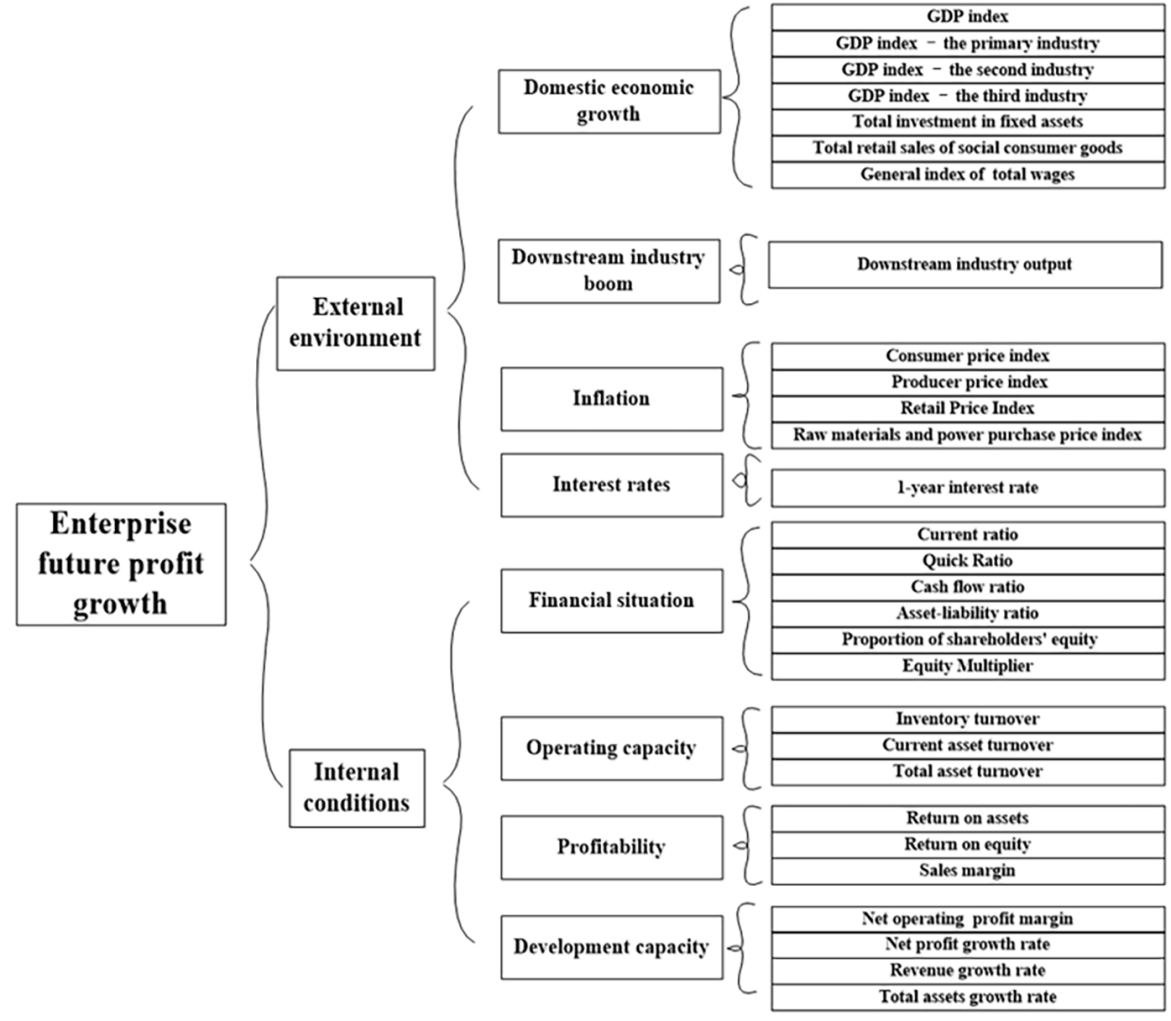
Evaluation factors.

Development of efficient methods to accurately estimate the company value requires assessing the conditions of the external and internal environment of the company. We consider the specific application environment for the products of each RFID company, for example, in the financial system, real estate, logistics, retail, product traceability, vehicle, railway, software system and other industries. Since downstream company usually purchases the large majority of its needs from the upstream company, downstream industry boom index is adopted to descript the demand scale of the downstream companies. The other external environment factors include domestic economic growth, inflation, and interest rate. And the factors depicting the internal conditions of the company consist of financial indicators which describe company's health survival and development capacity, company operation indicators that characterize business operating capacity, profitability indicators that represent the corporation profitability, and development capacity indicators that depict company potential ability for expanding scale and growing strength. The factor hierarchy structure shown in Fig 2 illustrates key factors which are said to impact on the company future profit growth. A total number of 29 indicators are considered, and all these 29 indicators can be further grouped into 8 dimensions. Once the key factors affected the profit are identified, we develop the value evaluation model which integrates these factors into a cause-effect framework, as shown in the following sections.

### 2.2. Evaluate the value of company’s worth based on ROA

There is uncertainty over the future rewards from the RFID investment, and these investments are partially or completely irreversible. ROA are most valuable when uncertainty is high, therefore we use ROA to calculate the company value, which is given by the following formula.

$$V=V_{a}+V_{c}$$ (1)

*s*.*t*.

$$V_{a}=\sum_{i=1}^{n}\frac{F_{i}}{{\left(1+r\right)}^{i}}+V_{s}$$ (2)

$$V_{c}=V_{S}N(d_{1})-Xe^{-rT}N(d_{2})$$ (3)

$$Y=V_{a}{\left(1+r\right)}^{T}$$ (4)

$$d_{1}=\frac{ln(\frac{V_{S}}{Y})+(r+\frac{1}{2}\sigma^{2})}{\sigma \sqrt{T}}$$ (5)

$$d_{2}=d_{1}-\sigma \sqrt{T},$$ (6)

where *Va* is the discounted value of real assets obtained by discounted cash flow method. *Vc* is the discounted value of future growth opportunities, *i*.e. the discounted value of options. *F*_*i*_ is the net profit at year *i* and obtained with ECMM, which is provided in next section. *r* is risk-free interest rate, *V*_*S*_ is the current value of the company, which is determined by tangible assets, such as currencies, buildings, real estate, vehicles, inventories, and equipment. *Y* is the final price of the company, *T* is the option expiration period, *σ* is the volatility of asset returns, *N*(*x*) is the cumulative probability distribution function of standard normal distribution.

## 3. Profit forecast based on ECMM

In this section, we present how to achieve low forecasting error by customizing graph construction and flexibilizing the net profit forecasting model based on ECMM.

### 3.1. Definition of NG

NG is formally defined as: *G* = (*W*; *E*), where *W* is the set of network nodes, *E* is the set of edges. Nodes denote independent or dependent variables. If two nodes appear in the same sample, an edge is drawn between them. We divide NG nodes into three categories: (a) evaluation factor node representing each evaluation factor, (b) classification node which denotes a grade of one evaluation factor, and (c) output node or clustering center node indicating one grade of output variable. Notice that each output node indicating the clustering center of one community. Each edge is assigned a weight by counting the number of its occurrences in dataset. An example of constructing a NG from the dataset shown in Table 1 is provided as follows.

**Table 1:** The dataset.

	*I*_1_	⋯	*I*_*H*_	*Z*_11_	*Z*_12_	⋯	*Z*_*HM*−1_	*Z*_*HM*_	*C*_1_	⋯	*C*_*J*_
*X*_1_	1		1	0	1		0	1	0		0
*X*_2_	1		1	0	1		1	0	1		0
⋮		⋮				⋮				⋮	
*X*_*N*_	1		1	1	0		0	1	0		1

**Fig 3:**
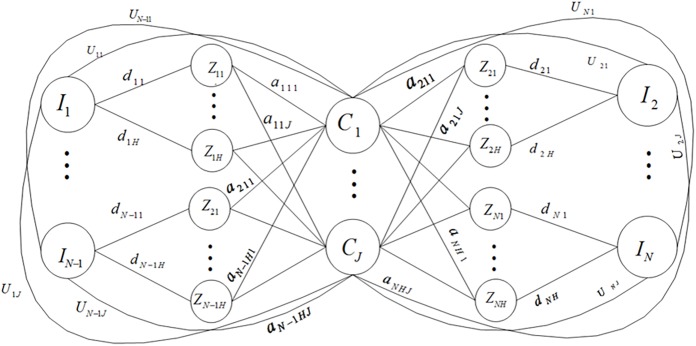
NG constructed based on the dataset.

In Table 1, *X*_*i*_ means sample *i*, *I*_*i*_ represents evaluation factor node *i*, *Z*_*ij*_ represents the *j*th classification node of evaluation factor *i*, similarly, *C*_*i*_ is the clustering center node and presents the *i*th grade node of output variable. In a sample, evaluation factor nodes, the classification nodes which indicates the value of each evaluation factor and the output node which denotes the sample output are indicated by 1, and other nodes are indicated by 0. Fig 3 shows NG generated from dataset in Table 1.

### 3.2. Clustering credibility and BPF

Basic measures of NG include: the node degree distribution, average path length, clustering coefficient, degree of correlation coefficient, betweenness, etc [17]. However, these measures usually indicate the structure features of NG, rather than expresses the clustering rules implied in network. In this study, we propose the index of clustering credibility, namely, the posterior probability of the node belonging to a community. The credibility index helps to discover the rules hidden in the network.

**Fig 4:**
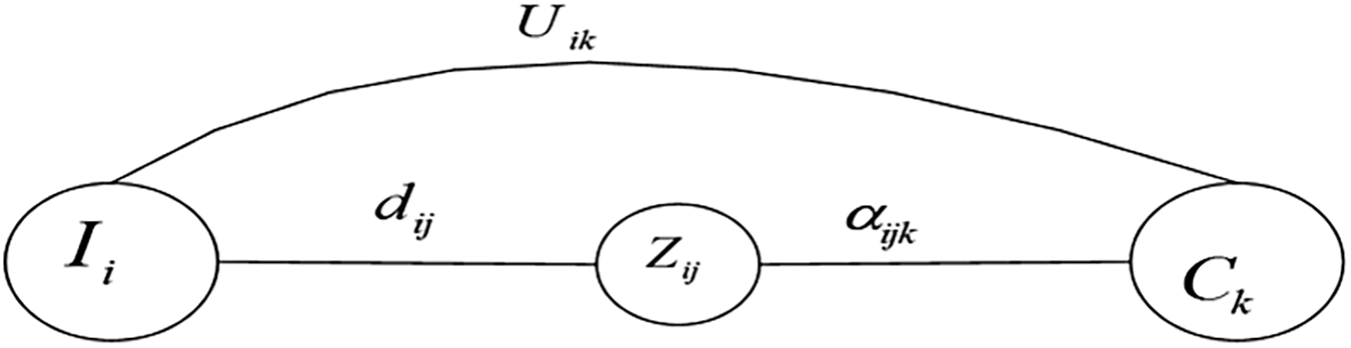
The unit of clustering rule in NG.

The basis unit of clustering rules of NG in Fig 3 can be represented as Fig 4, which indicates that: if the co-occurrence number of *I*_*i*_ and *C*_*k*_ is *U*_*ik*_, and that of *I*_*i*_ and *Z*_*ij*_ is *d*_*ij*_, the number of *Z*_*ij*_ belonging to community *C*_*k*_ is *α*_*ijk*_. In this paper, the main idea of CM is to determine whether node *Z*_*ij*_ can be allocated to community *C*_*k*_, but the clustering rule in Fig 4 still fails to intuitively judge the credibility of node *Z*_*ij*_ belongings to community *C*_*k*_. As a result, BPF is adopted to figure out the credibility of a node belonging to each community.

In Fig 4, the credibility of node *Z*_*ij*_ belongs to community *C*_*k*_ is mainly decided by parameter *U*_*ik*_, *α*_*ijk*_, and *d*_*ij*_. According to the definition of NG, we have $U_{ik}=\sum_{h=1}^{H}\alpha_{ihk}$. Therefore, the clustering credibility of node *Z*_*ij*_ is only determined by *α*_*ijk*_ and *d*_*ij*_. However, constrained by the number of RFID companies in real life, adequate samples cannot be collected. So *α*_*ijk*_ and *d*_*ij*_ obtained by the finite samples are not representative and only according to these two parameters, it is difficult to get the accurate clustering credibility of a node. Thus, we add offset *ε*_*ijk*_ and *β*_*ij*_ to *α*_*ijk*_ and *d*_*ij*_, respectively.

Accordingly, the posterior probability of node *Z*_*ij*_ belongs to community *C*_*k*_ is *P*(*C*_*k*_|*Z*_*ij*_;*d*_*ij*_;*α*_*ijk*_;*β*_*ij*_;*ε*_*ijk*_), and the estimated parameters are *ε*_*ijk*_ and *β*_*ij*_. As BM is suitable for the situation where the variables for classification are parametric variables, it is used to obtain this posterior probability [18]. To sample *X*, in order to judge its output, we need to calculate the posterior probability *P*(*C*_*k*_|*Z*_*ij*_;*d*_*ij*_;*α*_*ijk*_;*β*_*ij*_;*ε*_*ijk*_), then decide the community which *Z*_*ij*_ belongs to. According to Bayesian formula, we have

$$P(C_{k}|Z_{ij};d_{ij};\alpha_{ijk};\beta_{ij};\varepsilon_{ijk})=\frac{P(C_{k}|d_{ij};\alpha_{ijk};\beta_{ij};\varepsilon_{ijk})P(Z_{ij}|C_{k};d_{ij};\alpha_{ijk};\beta_{ij};\varepsilon_{ijk})}{P(Z_{ij}|d_{ij};\alpha_{ijk};\beta_{ij};\varepsilon_{ijk})}$$ (7)

The derived equation above shows that by observing the value of *P*(*C*_*k*_|*d*_*ij*_;*α*_*ijk*_;*β*_*ij*_;*ε*_*ijk*_) and *P*(*Z*_*ij*_|*C*_*k*_;*d*_*ij*_;*α*_*ijk*_;*β*_*ij*_;*ε*_*ijk*_), the posterior probability of node *Z*_*ij*_ belongs to community *C*_*k*_ can be converted to the posterior probability *P*(*C*_*k*_|*Z*_*ij*_;*d*_*ij*_;*α*_*ijk*_;*β*_*ij*_;*ε*_*ijk*_).

The maximum likelihood estimation of *P*(*C*_*k*_|*d*_*ij*_;*α*_*ijk*_;*β*_*ij*_;*ε*_*ijk*_) is shown as follows: $P(C_{k}|d_{ij};\alpha_{ijk};\beta_{ij};\varepsilon_{ijk})=\frac{\sum_{l=1,Z_{ij}\in X_{l}}^{\left|\psi \right|}P(C_{k}/Z_{ij})}{\left|\psi \right|}$, where *X*_*l*_ is training samples, *ψ* is the set of training samples, and |*ψ*| is the set size of *ψ*.

*P*(*Z*_*ij*_|*C*_*k*_;*d*_*ij*_;*α*_*ijk*_;*β*_*ij*_;*ε*_*ijk*_) is the class-conditional prior probability, and using the traditional method to obtain its expression is very difficult. Generally, if a node belongs to a community, the link density between it and the cluster center node of the community is high, otherwise the link density between these nodes is low. That is similar to the interaction force between particles in the electric field, namely the weak force among nodes result in low density and the high force among nodes leads to high density. Accordingly, *P*(*Z*_*ij*_|*C*_*k*_;*d*_*ij*_;*α*_*ijk*_;*β*_*ij*_;*ε*_*ijk*_) can be approximately defined by Gaussian potential function (GPF) which is mainly used to describe the interaction force of electric particles[19], as is shown in Eq 8.

$$P(Z_{ij}|C_{k};d_{ij};\alpha_{ijk};\beta_{ij};\varepsilon_{ijk})=e^{-\frac{d_{ij}+\beta_{ij}}{(\alpha_{ijk}+\varepsilon_{ijk})\theta }},$$ (8)

where the impact factor *θ* is used to adjust the influence boundary.

### 3.3. CM and profit forecast

After all the posterior probabilities of each classification nodes in a particular sample *X* being annotated to each category have been calculated, the overall probability for input sample *X* to be annotated to a particular category, *C*_*k*_ is calculated (i.e. *D*(*X*,*C*_*k*_)), which is shown in Eq 9.

$$D(X,C_{k})=\sum_{z_{ij}\in X}P(C_{k}|Z_{ij};d_{ij};\alpha_{ijk};\beta_{ij};\varepsilon_{ijk})$$ (9)

The Bayesian classifier is able to determine the right category of input sample *X* by the category which has the highest posterior probability value, as shown in Eq 10.

$$F_{X}=arg\;max\left(D\left(X,C_{k}\right)\right),\;k=1,2,\cdots ,j$$ (10)

## 4. IBFOA

In this section, the major components of IBFOA are provided, and IBFOA is developed to optimize the parameters of BPF, say, ε, β, and *θ*.

### 4.1. Bacterium encoding

**Fig 5:**
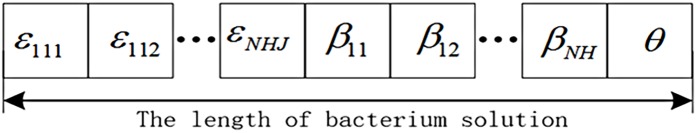
Bacterium coding diagram.

As shown in Fig 5, each bacterium solution mainly consists of three parts: the offset of edge weight between the classification nodes and cluster center nodes, the offset of edge weight among the classification nodes, and impact factor *θ* in Eq 8.

### 4.2. Assess multiple objectives based on PBM

In order to improve the prediction reliability of the algorithm, we optimize two objectives, i.e. MSE and ME, simultaneously. We utilize versatile computational intelligent models such as PBM for handling this type of problems. MSE and ME is shown in Eq 11 and Eq 12, respectively.

$$MSE=\frac{1}{n}\sqrt{\sum_{i\in \Omega }{\left(F_{i}-{\hat{F}}_{i}\right)}^{2}},$$ (11)

$$ME=\underset{i\in \Omega }{max}\{\left|F_{i}-{\hat{F}}_{i}\right|\},$$ (12)

where Ω represents the set of all prediction samples, *F*_*i*_ is the actual output of sample *i*, ${\hat{F}}_{i}$ is the predicted output of sample *i*, *n* is the number of prediction samples.

Next, we show how to integrate MSE and ME into one objective function according to PBM for the sake of converging into optimal solutions and maintaining the nature of uniform distribution among solutions.

Define that as long as MSE or ME of an solution is not dominated by others, the solution is not dominated by others, that is, given population *P*, for *p*, *q*∈*P*, if *MSE*(*p*) < *MSE*(*q*) or *ME*(*p*) < *ME*(*q*), then *p* is not dominated by *q*. Then the main steps of ranking solutions using single non-dominated sorting method (SNDSM) are shown as follows:

1. All solutions are sorted in ascending order based on MSE and ME, respectively. Then the rank of solution *q* (∀*q*) for each indicator is obtained, namely S_MSE_(*q*) and S_ME_(*q*).
2. Select the lower rank of the solution in two objectives, that is, min{*S*_*MSE*_(*q*),*S*_*ME*_(*q*)}. In the same way, the entire population is graded.

**Fig 6:**
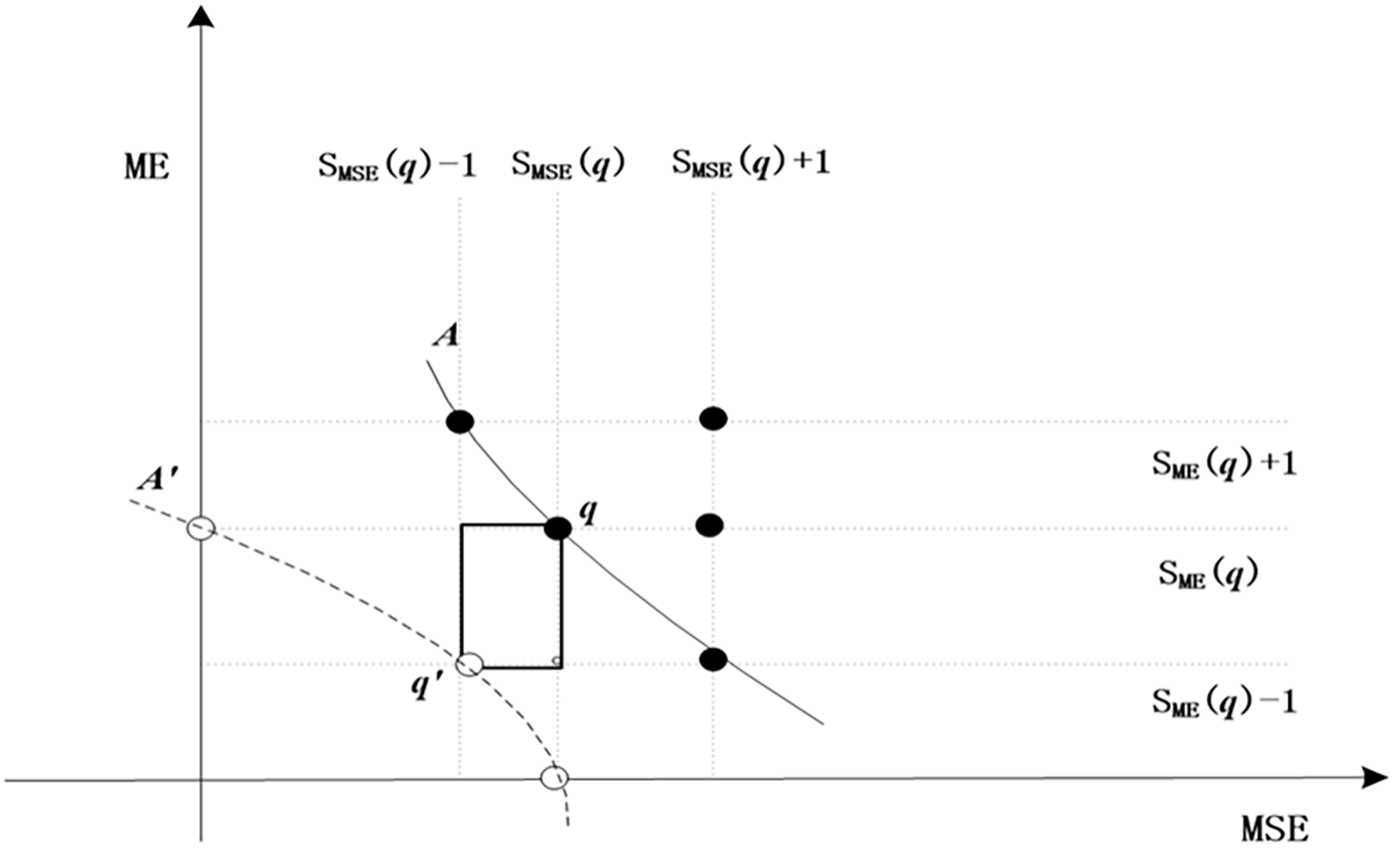
The example of SCD.

In fact, relying solely on the above SNDSM still cannot sort solutions in the same grade set, we call these solutions undetermined solutions (USs), and we further rank them according to the single congestion degree (SCD). Fig 6 shows the example of SCD. Define the ultra-optimized solution for each solution in US set, whose rank of each objective is immediately lower than the solution, for instance q' is the ultra-optimized solution of q. The boundary consisting of those ultra-optimized solutions are called ultra-optimized boundary, namely A'. SCD is proposed to measure the closeness between a solution and its ultra-optimized solution. Intuitively, SCD is the perimeter of the rectangle in Fig 6, which is composed of the solution point (i.e. q) and its ultra-optimized solution point (i.e. q') on the ultra-optimized boundary. For a solution in US set, the less SCD means more optimal, and result in lower ranking.

### 4.3. Chemotaxis operation

Unlike the fixed-parameter bacterial foraging optimization algorithm (BFOA), in the chemotaxis operation of IBFOA, the swim step is adjusted according to the solutions’ objective values adaptively. The detailed chemotaxis operation is described like this:

1. Tumble operation. A unit length random direction, say vector Δ(*i*) ∈ [−1,1], is generated for each bacterium and this defines the direction of movement after a move. The update of bacteria is mainly according to Eq 13.
  $$Q(i,t+1,k)=Q(i,t,k)+H(t,k)\frac{\Delta (i)}{\sqrt{\Delta {\left(i\right)}^{\text{T}}\Delta (i)}},$$ (13)
  where *Q*(*i*,*t*,*k*) is the code of the *k*th position of bacterial *i* at iteration *t*, *H*(*t*, *k*) is the step size of direction for position *k*, which is generated randomly.
2. Swim operation. If the better solution is found in tumble operation, *H*(*t*, *k*) is updated in the generated direction, as shown in Eq 14, and continue to create bacteria until no better ones are found.
  $$H(t+1,k)=\min\{\frac{MSE(i)-MSE_{best}}{MSE_{\max}-MSE_{\min}}H(t,k),\frac{ME(i)-ME_{best}}{ME_{\max}-ME_{\min}}H(t,k)\},$$ (14)
  where *MSE*_*best*_ is the best MSE value found so far and *ME*_*best*_ is the best ME value.

### 4.4. Reproduction operation

In reproduction operation, half of the optimal bacteria reproduce their offspring. Specially, we rank each bacterium according to PBM and half of bacteria with poor ranking reproduce. Assuming that every component in bacteria is independent with each other, and follow the Gaussian distribution, the reproduction operation presented as follows:

$$Q_{t+1}=\eta_{norm}\sigma_{t}+\mu_{t},$$ (15)

$$\eta_{norm}=\sqrt{-2\ln\eta_{1}}\cos(2\pi \eta_{2}),$$ (16)

where *η*_1_ and *η*_2_ are the uniformly distributed random number in interval [0, 1], the formula generating *η*_*norm*_ is called BOX-Muller formula, *μ*_*t*_, *σ*_*t*_ are the mean and standard deviation of the element in each position of the bacteria generated in iteration *t* and before.

### 4.5. Elimination-dispersal operation

In traditional BFOA, the elimination-dispersal probability for each bacterium solution is equal [20]. However, that will lose potential Pareto optimal solutions, consequently IBFOA sets elimination-dispersal probability mainly according to the PBM ranks of bacterium, shown as follows:

$$S_{i}=\frac{R_{i}}{R_{\max}}P_{ed},$$ (17)

where *R*_max_ is the maximum rank according to PBM, *R*_*i*_ is the rank of bacterium *i*, *P*_*ed*_ is the basic elimination-dispersal probability.

### 4.6. IBFOA procedure

A complete and detailed IBFOA pseudo codes are provided in the following.

1. Generate initial population, initiate the parameters of IBFOA.
2. Evaluate the population
  Calculate ME and MSE.
3. Use PBM to rank the population.
4. Chemotaxis operation.
5. Reproduction operation.
6. Elimination-dispersal operation.
7. Evaluate the new solutions generated from step 4 to 6.
  Calculate ME and MSE.
8. Rank the population:
  - Rank all solutions using PBM
  - Reserve solutions ranked in the front
9. If the end condition is satisfied then stop, otherwise go to step 4.

## 5. Experiment design

The application of the proposed model in solving a real world business valuation problem by determining the community structure in NG is presented in this section. Performance assessment is made in terms of forecast accuracy, running time for optimal solutions and domain knowledge extraction.

### 5.1. Experimental setup and baseline methods

We collected data on 13 typical Chinese RFID listed companies, and their main business areas covered are: tags and packaging, reading and writing equipment, system integration and software development. This data set contains samples of these companies from 1997 to 2014. Removing invalid samples, and 155 short-term (one-year-ahead forecast) samples, 142 medium-term (two-year-ahead forecast) samples and 129 long-term (three-year-ahead forecast) samples were obtained. We scaled the collecting data into interval [1, 5].

We compared the performance of our method with several existing methods: RMs, support vector machine (SVM), NNs, NSGAII and SPEA2, which are two highly competitive algorithms for bi-objective optimization. We adopted 4 most commonly used RMs: multiple curvilinear regression with the kernel function of *y* = *ax*^*b*^ (RM1), $y=\frac{1}{a+be^{-x}}$ (RM2), *y* = *a*+*bx*+*cx*^2^ (RM3), and *y* = *a*+*bx*+*cx*^2^+*dx*^3^ (RM4). Three kinds of NNs were adopted: back propagation NN (BPNN), general regression NN (GRNN), and radial basis function (RBF) NN. The reasons for selecting these NNs to present a contrast is that: BPNN is the most widely used NN, GRNN has the ability to converge to the underlying functions of the data with only few training samples available and needs relatively small knowledge to turn the parameter, and RBFNN has the characteristics of best approximation and global optimal performance.

IBFOA, NSGAII and SPEA2 were adopted to find the community and further forecast the net profit. The proposed IBFOA was implemented in VB. SVM, NNs, and RMs aimed at optimizing MSE and were achieved in Matlab with the default setting. NSGAII and SPEA2 were also designed in Matlab environment according to the codes provided in [21] and [22], respectively. The population size of NSGAII and SPEA2 were 20 and both ran 100 iterations.

In IBFOA, initiated *β* and *ε* between -8.25~8.25, initiated *θ* between 0~1, set elimination-dispersal probability (*Ped*) to be 0.9, and the population size to be 20, IBFOA was run 100 iterations. In ROA models, risk-free interest rate *r* was replaced by the bank’s benchmark interest rate, which was selected as 3.5%, σ was set to be 40%.

### 5.2. Performance of ECMM

The valuation deviation at year *t* is calculated as follows:

$\Delta V(t)=\frac{1}{n}\sum_{i\in \Omega }{\left(V_{i}(t)-{\hat{V}}_{i}(t)\right)}^{2}$, where *V*_*i*_(*t*) is the actual value of sample *i*, ${\hat{V}}_{i}(t)$ is the predicted value, and *n* is the total number of test samples.

**Fig 7:**
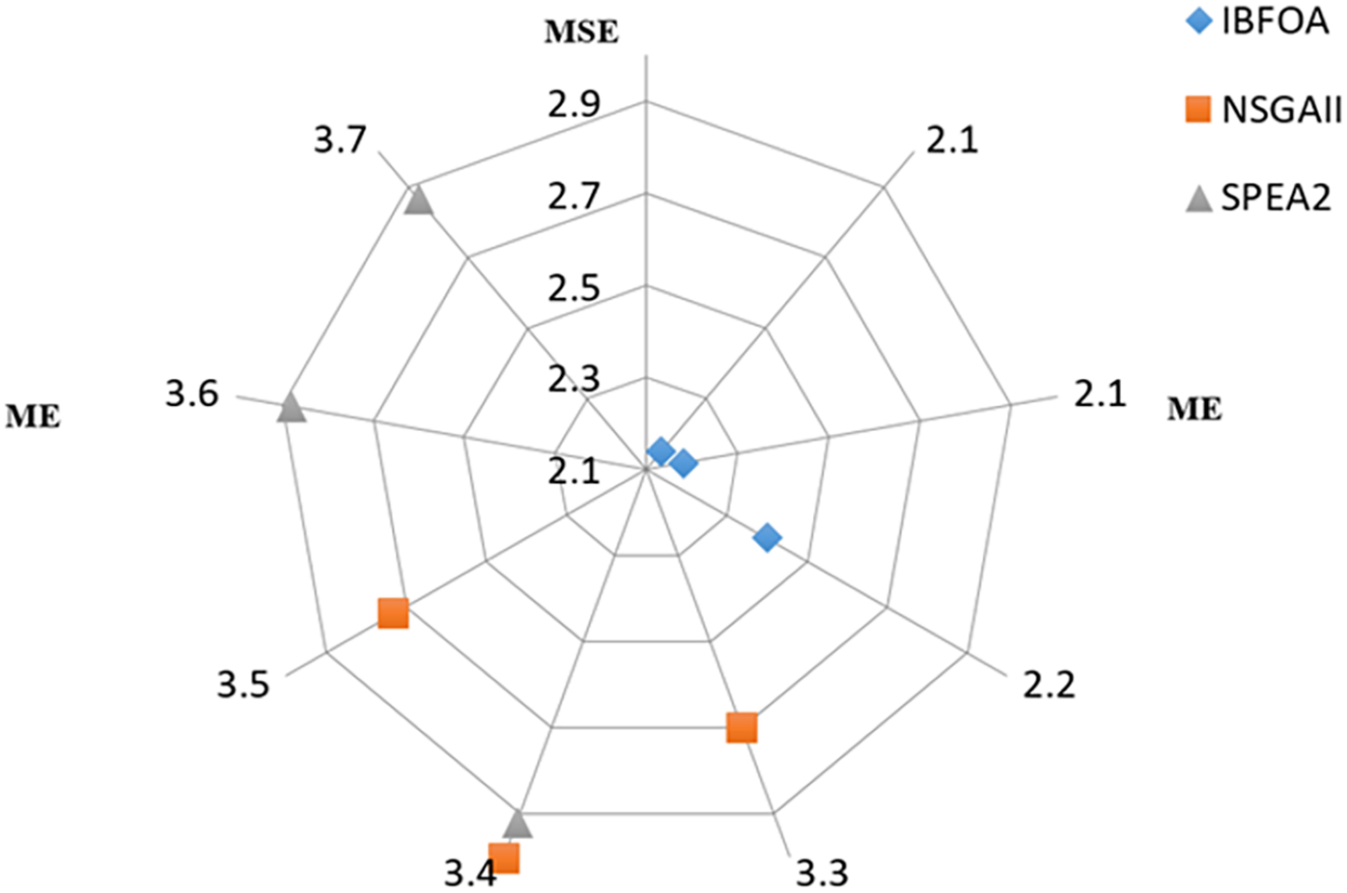
The results of bi-objective algorithms.

Three types of experiments were carried out: short-term, middle-term, and long-term test. In each test, randomly selected 100 samples as training samples and others as testing samples. The ME, MSE of profit forecast and ∆V are shown in Tables 2–4, respectively, where AV1 and UB1 represent average value (AV) and upper bound (UB) of ME and MSE in short-term test, AV2 and UB2 indicate that in medium-term test, and AV3 and UB3 mean that value of long-term test. Fig 7 shows the radar chart of average value of ME and MSE in different terms for various bi-objective algorithms.

**Table 2:** The experiment results of MSE.

	BP	GRNN	RBFNN	RM1	RM2	RM3	RM4	SVM	SPEA2	NSGAII	IBFOA
AV1	3.39	3.33	3.44	2.96	3.62	3.66	4.93	4.88	2.87	2.70	**2.15**
UB1	4.51	4.16	6.82	3.71	4.76	4.89	6.33	6.24	3.45	3.67	**2.29**
AV2	3	3.35	5.46	2.95	3.2	3.68	4.6	4.39	2.88	2.73	**2.18**
UB2	4.21	4.14	6.9	3.81	3.88	4.88	5.9	6.05	4.16	4.47	**2.40**
AV3	2.89	3.41	5.26	2.99	3.33	3.73	4.59	4.55	2.92	3.0	**2.40**
UB3	5.03	4.48	7.76	3.93	4.03	4.55	6.03	5.69	4.13	3.96	**2.93**

**Table 3:** The experiment results of ME.

	BP	GRNN	RBFNN	RM1	RM2	RM3	RM4	SVM	SPEA2	NSGAII	IBFOA
AV1	3.4	3.9	2.3	3.8	4	4	4	4	3.7	3.3	**2.1**
UB1	4	4	4	4	4	4	4	4	4	4	**3**
AV2	3.3	3.9	3.8	3.4	3.8	4	4	4	3.6	3.5	**2.1**
UB2	4	4	4	4	4	4	4	4	4	4	**3**
AV3	3.1	3.8	3.8	3.4	3.7	4	4	4	3.4	3.4	**2.2**
UB3	4	4	4	4	4	4	4	4	4	4	**3**

**Table 4:** The experiment results of ∆V.

	BP	GRNN	RBFNN	RM1	RM2	RM3	RM4	SVM	SPEA2	NSGAII	IBFOA
AV1	3.27	3.21	3.21	2.85	3.49	3.53	4.76	4.71	2.77	2.60	**2.07**
UB1	4.35	4.01	6.58	3.58	4.59	4.72	6.11	6.02	3.33	3.54	**2.21**
AV2	5.57	6.04	6.95	5.65	6.9	7.07	9.25	7.94	5.02	4.87	**3.86**
UB2	7.65	7.19	15.61	7.54	8.55	9.67	12	12.15	5.93	6.24	**5**
AV3	7.74	8.48	18.03	13.02	15.54	16.29	20.86	17.4	7.9	7.83	**6.32**
UB3	12.1	10.65	32.97	19.42	20.24	22.52	27.52	24.44	10.44	10.31	**9.96**

As can be seen from Tables 2–4, whether in the test of net profit forecast or company value evaluation, our IBFOA demonstrates superior performances, namely MSE, ME and ∆V are the smallest. Those show that using IBFOA to optimize BPF and clustering NG with BPF are good at predicting the profit of company and ECMM is expert in evaluating the business value.

In Fig 7, the solutions of IBFOA densely distribute in the internal and optimal area of the radar chart, while the solutions of NSGAII and SPEA2 uniformly disperse in the outer edge of the chart. Therefore, the solutions of IBFOA are more Pareto optimal than those of NSGAII and SPEA2, in other words, NSGAII and SPEA2 are dominated by IBFOA, and those mean that the proposed IBFOA contributes to promoting the convergence of the algorithm as well as maintaining the diversity of the population.

### 5.3. Running time for obtaining optimal solutions

By the mechanism of IBFOA, we can obtain its time complexity O(n^2^), where *n* is the length of bacterium solution, as shown in Fig 5. Table 5 shows the running time of various models for each experiment. In Table 5, compared with other models, although our IBFOA takes a little more time, its high precision offsets this deficiency.

**Table 5:** Runtime comparison for all models.

	BP	GRNN	RBFNN	RM1	RM2	RM3	RM4	SVM	SPEA2	NSGAII	IBFOA
Time	<15s	<15s	<15s	<15s	<10s	<10s	<10s	<10s	≈30s	≈30s	≈**40s**

### 5.4. Domain knowledge extraction

We run IBFOA 100 iterations on the collected dataset to acquire the optimal offsets of the edge weight in NG, and then calculate the weights of nodes. Specifically, in Fig 3, for any evaluation factor node *I*_*i*_, its weight is the sum of the absolute value of weight *d*_*ij*_ and its offset β_*ij*_, that is, $\sum_{j}\left|\beta_{ij}+d_{ij}\right|$, similarly, for any classification node *Z*_*ij*_, its weight is $\sum_{k}\left|\alpha_{ijk}+\varepsilon_{ijk}\right|$. Then nodes are sorted in descending order of their weights, and top 10 nodes of each type are displayed in Table 6.

**Table 6:** Top 10 nodes of each type.

Rank	Factor node	Classification node
1	Net profit growth rate	GDP, grade 5
2	Revenue growth rate	General index of total wages, grade 3
3	Producer price index	1-year interest rate, grade 1
4	1-year interest rate	Total retail sales of social consumer goods, grade 1
5	GDP index–the second industry	Current asset turnover, grade 4
6	Sales margin	Raw materials and power purchase price index, grade 4
7	GDP	Producer price index, grade 5
8	Consumer price index	Return on assets, grade 4
9	Total investment in fixed assets	Total assets growth rate, grade 2
10	Net operating profit margin	Total asset turnover, grade 1

As can be seen from Table 6, factors significantly affecting the profitability of companies are net profit growth rate, revenue growth rate, producer price index, interest rate, etc. Although some factors, such as general index of total wages, total retail sales of social consumer goods, current asset turnover, and so on, are not listed as the key evaluation factors, some of their classification nodes have a significant impact on profitability. This shows that the NG based method can not only depict which factors have an effect on the profitability of companies, but also can describe the specific impact of each value of factors, for example, “GDP, grade 5” in Table 6 indicates if the GDP factor value is 5, it has an important impact on profitability.

## 6. Conclusions and future works

The valuation of the company is the basis for determining the relevant negotiating parameters when venture capitalist and entrepreneur negotiate the deal. A reliable valuation model helps to provide the basic standard for the measurement of the company’s fair market value as well as determining its price. In this study, combing CM method and ROA, a novel business valuation model for RFID companies is proposed. Compared with the existing methods, the proposed technique is distinctive in the following aspects:

Firstly, unlike the current RM or NN based forecast methods, our method is based on NG. Moreover, we calculate the credibility of a node belonging to each community of NG, and then cluster the network based on the credibility. That is different from the traditional CM methods, which cluster the network directly using the network structure.

Secondly, different from current BM which adopts rigid and fixed function expressions, in this study, the flexible BM is developed to calculate the clustering credibility, that is, adopt GPF to approximate BPF and use IBFOA to optimize the parameters of BPF.

Thirdly, dissimilar to general single objective BFOA, IBFOA is bi-objective algorithm, which uses PBM to evaluate the reliability of forecast model. In the proposed PBM, SNDSM and SCD are adopted to rank the solutions, which can keep the diversity of the solutions without the cost of delaying the algorithm convergence.

Finally, in IBFOA, new solutions are generated mainly through integrating the merits of existing solutions, and that spurs further search along the optimal direction so as to promote the convergence of the algorithm. Simultaneously, the parameters of the algorithm are adaptively adjusted according to the performance of IBFOA to further improve its efficiency.

Simulation results of RFID companies show that the proposed model demonstrates higher accuracy and reliability compared with other models. Future research will focus on the further improvement of the proposed model of NG. Although the experiment results show NG structure in this paper is appropriate, we cannot prove that it is optimal. So an optimal NG structure for business valuation is hoped to be given in future studies.
